# Longitudinal association between exposome score for schizophrenia and clinical features: results from the Athens First-Episode Psychosis Research Study

**DOI:** 10.1192/j.eurpsy.2022.1963

**Published:** 2022-09-01

**Authors:** G. Erzin, L. Pries, S. Dimitrakopoulos, I. Ralli, L.-A. Xenaki, R.F. Soldatos, I. Vlachos, M. Selakovic, S. Foteli, I. Kosteletos, N. Nianiakas, L. Mantonakis, E. Rizos, K. Kollias, J. Os, S. Guloksuz, N. Stefanis

**Affiliations:** 1 Ankara Dışkapı Training and Research Hospital, Psychiatry, ANKARA, Turkey; 2 Maastricht University Medical Center, Department Of Psychiatry And Neuropsychology, School For Mental Health And Neuroscience, maastricht, Netherlands; 3 National and Kapodistrian University of Athens Medical School, Eginition Hospital, First Department Of Psychiatry, Athens, Greece; 4 National and Kapodistrian University of Athens Medical School, Eginition Hospital, First Department Of Psychiatry, Athens, Greece; 5 National and Kapodistrian University of Athens Medical School, Second Department Of Psychiatry, Athens, Greece

**Keywords:** environmental exposures, clinical features, exposome, Psychosis

## Abstract

**Introduction:**

Previously, environmental vulnerability for schizophrenia assessed through exposome score for schizophrenia (ES-SCZ) was associated with the risk for psychosis development.

**Objectives:**

The current study aims to investigate the longitudinal association between ES-SCZ and symptom severity in individuals with first episode psychosis (FEP) to understand how environmental exposures affect illness course.

**Methods:**

Baseline and 1-month follow-up assessments were available for 225 individuals with FEP from the Athens FEP Research Study. The Positive and Negative Syndrome Scale (PANSS) was used to measure clinical features. In accordance with previous reports, the ES-SCZ was calculated by summing log-odds weighted environmental exposures (childhood adversities, winter birth, and cannabis use). To model the course of clinical features over time the effects of the ES-SCZ-by-time interaction, ES-SCZ, and time were analyzed with multilevel regression analyses. Age, sex, and education were added as covariates

**Results:**

The analyses of change of PANSS total score over time indicated that clinical features decreased from baseline to the 1-month follow-up assessment. The association between ES-SCZ and PANSS total score were not statistically significant. The analyses of the PANSS total score over time indicated an ES-SCZ-by-time interaction (B = 2.82 [95% CI 0.28; 5.35], P-value = 0.029), meaning the decrease of the PANSS total score over time were dependent on ES-SCZ and individuals with high ES-SCZ showed less improvement

**Conclusions:**

The findings show that the total environmental predisposition to schizophrenia (ES-SCZ) not only increases the risk for psychosis development but may also influences the illness course.

**Disclosure:**

No significant relationships.

